# Regulatory focus, coping strategies and symptoms of anxiety and depression: A comparison between Syrian refugees in Turkey and Germany

**DOI:** 10.1371/journal.pone.0206522

**Published:** 2018-10-25

**Authors:** Karl-Andrew Woltin, Kai Sassenberg, Nihan Albayrak

**Affiliations:** 1 Department of Psychology, University of Roehampton, London, United Kingdom; 2 Leibniz-Institut für Wissensmedien, Tübingen, Germany; 3 University of Tübingen, Tübingen, Germany; 4 Department of Psychological and Behavioural Science, London School of Economics and Political Science, London, United Kingdom; University of New South Wales, AUSTRALIA

## Abstract

Civil war, flight, escape and expulsion are extremely stressful and assert a negative impact on refugees’ mental health. However scientific research about resilience and coping of refugees is scarce. Especially in the recent refugee crisis, calls have been made to consider factors contributing to coping and resilience in this vulnerable population. Therefore, the current research sought to investigate individual differences that could serve as antecedents of coping and contextual factors that might moderate these effects. Specifically, it took into account individual’s self-regulatory differences in terms of regulatory focus (i.e., a promotion focus on nurturance needs, ideals and gains vs. a prevention focus on security needs, oughts and losses). It furthermore explored contextual influences by considering Syrian refugees in Turkey (Sample 1, *N* = 273) and Germany (Sample 2, *N* = 169). Compared to Syrian refugees in Turkey, those in Germany had a stronger promotion focus. They also reported more problem-focused and less maladaptive coping, as well as less symptoms. Both promotion and prevention focus were positively related to problem-focused coping. Problem-focused coping, in turn, predicted more symptoms in Turkey but not in Germany. Furthermore, a stronger promotion focus was associated with less symptoms and maladaptive coping was associated with more symptoms in both samples. These results contribute to the coping literature in demonstrating that under certain conditions problem-focused coping can be maladaptive and extend the scarce previous work on self-regulation and coping. Most importantly, they highlight a promotion focus as a clear resilience factor and the role of maladaptive coping in increasing vulnerability. As such, they might inform the design of effective interventions among Syrian refugees and beyond.

## Introduction

The United Nations refugee agency reported that in 2016, 65.6 million people–or about 1 percent of the world’s population–were refugees (i.e., forced displaced people; [1]). The Syrian conflict alone has forced over 12.5 million peoples (six out of 10 of the country’s prewar population) from their homes, making this one of the largest refugee displacements in recent history [2]. Pre- and post-displacement factors associated with mental health of refugees include multiple human rights violations and abuses, primary and secondary trauma, loss and concerns about family members, limited access to basic commodities, and problems caused by cultural differences, racism and isolation [3–5]. Consequently, emotional distress in refugees persists even years and decades after resettlement [6, 7]. The most important and clinically significant problems among Syrian refugees are symptoms of emotional distress related to depression, prolonged grief disorder, posttraumatic stress disorder and various forms of anxiety disorders [8, 9]. Prominent factors determining whether psychosocial problems or emotional distress result in mental disorders are refugees’ resilience and coping mechanisms [10, 11]. However, research on these factors is scarce regarding the mental health of refugees in general and Syrian refugees more specifically, leading to calls to take them into account to better inform programs that enhance functionality and coping strategies [8, 9, 12–15].

The current work responds to these calls by exploring critical factors. Specifically, it considers (a) individual differences in self-regulation (i.e., regulatory focus; [16]) as an antecedent of coping and mental wellbeing and (b) contextual influences by investigating their relations among Syrian refugees in Turkey and Germany–countries that provide a shelter to a large number of refugees, but differ not only in terms of support structures and perspectives, but also in their proximity to the conflict zone. As such, it is one of very few social psychological investigations among this highly vulnerable population [17] and aims to make a modest contribution to this urgent problem.

### Coping strategies and individual differences

Coping has been defined as cognitive and behavioral efforts that are put into place by people to manage specific external and/or internal demands that are appraised as taxing or exceeding their resources [18]. Over 400 categories or classifications of people’s ways of coping with life-problems (including behaviors, cognitions, and perceptions) exist in the literature [19]. This is due to coping being an organizational construct, encompassing the various actions individuals use to deal with stressful experiences, rather than a specific behavior or belief [20]. Consequently, different lower-order coping categories (e.g., seeking support, emotion expression, denial) have been organized into different higher-order coping categories (e.g., primary vs. secondary vs. relinquished control coping; engagement vs. disengagement coping) by different researchers [20, 21]. A widely applied and accepted conceptualization distinguishes problem-focused coping (i.e., dealing directly with the stressor to remove it or diminish its impact) from emotion-focused coping (i.e., dealing with the associated feelings of distress to minimize and manage them, e.g. using reappraisals; [18]). Building on this, the COPE inventory was developed [22] to assess problem-focused (e.g., active coping, planning), emotion-focused (e.g., positive reinterpretations, acceptance), and less useful, maladaptive coping responses. The latter includes not dealing with either the stressor or its associated distress by avoiding acknowledgement of the problem (e.g., denial; substance use), or by giving up the attempt to do anything about it (e.g., mental or behavioral disengagement). This three-fold distinction also broadly corresponds to coping dimensions in the common-sense model of self-regulation, in which individuals’ lay representations of illness stressors have been found to have direct and, via coping, indirect effects on health outcomes [23, 24]. The current work uses this prominent three-fold distinction of coping strategies affecting wellbeing [20], as has previous work on self-regulation and coping [25]. Meta-analyses found problem- and (with variations also) emotion-focused engagement coping to relate to better, and maladaptive disengagement coping to worse physical and mental health [26, 27].

However, physical and mental health under stress does not only depend on the applied coping strategies. Lazarus and Folkman’s [18] understanding of coping comprises a person-environment interaction: Different environments/stressors lead to variations in coping, and different people vary in their predispositions to cope in particular ways [24]. Indeed, differences in personality and self-regulation impact coping [21, 28]. An important individual difference is optimism, which is positively associated with problem- and emotion-focused coping, but negatively associated with maladaptive coping [29]. In a meta-analytic review the optimism-coping relationship was more substantial for engagement (positive relations with problem- and emotion-focused coping) than disengagement (negative relation with responses such as denial and venting, and thus maladaptive coping; [30]). Optimism is understood to be functional and adaptive because it sustains coping and wellbeing in times of stress. For example, optimist undergraduates were more likely than pessimists to use problem-focused coping, which in turn related to better adjustment in settling into college; they were also less likely to use maladaptive coping [31]. The relation between optimism and better mental health outcomes is thus at least partially mediated by differences in coping strategies. The concept of optimism may be somewhat misplaced when studying Syrian refugees who only recently experienced traumatic loss. However, there is a self-regulation strategy (i.e., a mindset in which goal striving is approached) that constitutes a motivational basis of optimism, namely regulatory focus, and more precisely a promotion focus [25, 32].

### Regulatory focus

Regulatory focus theory [16] distinguishes two distinct self-regulatory systems operating within individuals: a promotion and a prevention focus. The promotion focus regulates nurturance needs and is concerned with growth, advancement, and accomplishments. Individuals’ goals in a promotion focus are wishes and aspirations (ideals), and they pursue them using eager strategies, focusing on the presence/absence of positive outcomes (gains). Conversely, the prevention focus regulates security needs and is concerned with safety and responsibilities. Individuals’ goals in a prevention focus are duties and obligations (oughts), and they pursue them with vigilant strategies, focusing on the presence/absence of negative outcomes (losses; [33–35]). As a consequence of these different frames (gain vs. loss) and strategic inclinations (eagerness vs. vigilance), individuals in a promotion focus are motivated by upward counterfactuals (comparing the current to a better reality). In contrast, individuals in a prevention focus are motivated by downward counterfactuals (comparing the current to a worse reality; [36, 37]). Furthermore, a promotion focus is positively associated with optimistic forecasts, and optimistic forecasting increases engagement and persistence among promotion-focused individuals. Contrary, a prevention focus is positively associated with pessimistic forecasts, and pessimistic forecasting increases engagement and persistence among prevention-focused individuals [32]. Finally, optimism itself is positively associated with a promotion focus [25].

Because regulatory focus affects both how people appraise the world and their behavioral strategies in it, it is likely to influence coping behaviors and thus adjustment, especially in high demand situations when people’s self-regulatory system is stressed [28]. For example, when facing a demanding task, a promotion focus results in experiencing more challenge and less threat than a prevention focus, due to more perceived resources [38]. However, whilst some research explored differences in regulatory focus when coping with failure, self-control conflicts and intergroup interactions [28], studies directly targeting both coping strategies and regulatory focus are scarce, and inexistent concerning highly taxing and severe situations refugees are confronted with. To our knowledge, only one study directly assessed both regulatory focus and the coping strategies (using the COPE inventory) in relation to mental health (assessed with the Hopkins Symptoms Checklist, HSCL; [39]) and measured dispositional optimism [25]. In this research, promotion was positively related to optimism. Both foci were negatively related to anxiety and depression, positively related to aspects of problem-focused coping, negatively related to aspects of maladaptive coping, and unrelated to aspects of emotion-focused coping. Finally, optimism partially mediated the promotion-psychological symptoms link. However, this research left unclear what–presumably mundane–stressors undergraduate participants were coping with, only reported results for some COPE subscales, and did not report results for the indirect effects of regulatory focus on wellbeing via coping strategies, of interest in the current work. Nonetheless, based on this research regulatory focus, and especially promotion focus, should likewise be an antecedent of coping and in turn affect mental wellbeing (i.e., directly and indirectly via coping; [24]) among refugees.

However, contextual influences are likely to modify the above reported effects. The summarized research above was conducted in WEIRD countries (i.e., Western, educated, industrialized, rich, and democratic nations; [40]) providing a very different context from the one faced by the refugee groups considered here. Furthermore, and regarding the functionality of coping strategies, a comprehensive review of the literature concluded that it is impossible to determine the adaptiveness or maladaptiveness of any particular way of coping as this is ultimately determined by the specific stressor and situational constraints [20]. For example, faced with an uncontrollable stressor–a frequent experience for refugees–it may be adaptive to disengage [41].

### Contextual considerations and overview on predictions

Among different groups of Syrian refugees contexts and experiences are likely to differ substantively. An important contextual factor impacting wellbeing is closeness to the conflict zone, with refugees closer to the Syrian border reporting more distress [42]. It can thus be expected that conflict zone proximity will influence the use of the coping strategies. Likewise, the effects of regulatory focus on coping strategies and in turn on wellbeing might differ compared to earlier research due to such contextual differences.

The current work explores critical factors in coping and mental wellbeing of refugees. It builds on the notion of regulatory focus influencing coping and in turn adjustment in Western/WEIRD samples [28]. Going beyond previous research [25] it explores direct and indirect relations between regulatory focus, coping strategies, and mental wellbeing in Syrian refugees near (Sample 1, Turkey) and distal to the conflict zone (Sample 2, Germany). As such it makes several contributions. First and foremost, it responds to recent calls regarding the importance of investigating refugees’ coping and resilience [8, 9, 12–15]. Second, and relatedly, by taking into account individual differences in self-regulation it might inform more targeted interventions [21], providing an empirical basis to help design intervention programs. Third, considering samples in Turkey and Germany allows exploring the impact of conflict zone proximity on coping with the same stressor. Finally, it extends previous work considering undergraduate students and mundane stressors [25], thus allowing for a comparison with more severe stressors.

Several tentative predictions guided this research, based on the literature reviewed above. *First*, promotion (and to a lesser extent also prevention) should be positively related to problem-focused coping and in turn to better mental wellbeing [25] (Prediction 1, mediation; promotion and prevention should have an indirect effect via problem-focused coping on mental wellbeing). However, this relation might be stronger and perhaps only emerge in the Germany sample, as conflict zone proximity (along with other contextual factors) may lead to more distress and the situation being perceived as less controllable, thus undermining the adaptiveness of problem-focused coping in the Turkey sample [20, 24, 41, 43]. (Given the contradictory or null-findings regarding the effects of emotion-focused coping and its relation to regulatory focus and wellbeing, we did not deem it appropriate to formulate directed predictions). *Second*, in both samples promotion and prevention should be negatively related to maladaptive coping and in turn to better mental wellbeing [25] (Prediction 2, mediation; promotion and prevention should have an indirect effect via maladaptive coping on mental wellbeing). *Third*, conflict zone proximity can be expected to impact both the usage of the coping strategies as well as overall wellbeing [24, 42, 44] (Prediction 3, moderated mediation; the effects of promotion and prevention via problem-focused and maladaptive coping should be moderated by sample differences).

## Materials and methods

### Sample 1

#### Participants

Two hundred ninety-two Syrian refugees living in nine refugee camps near the Syrian border in Turkey participated in this study on a voluntary basis and were recruited with the help of a humanitarian organization. The humanitarian worker approached the refugees in Arabic, informed them about the content of the study, the right to withdraw at any time, and the anonymous treatment of the data. Refugees who were interested in taking part provided written informed consent before being handed the questionnaires. Data collection for both samples was approved by the ethics committee of Royal Holloway, University of London (i.e., the university the first and third authors were affiliated with at the time of data collection).

Data was collected over a period of two months in the summer of 2015. After excluding participants with very high numbers of missing values the final sample comprised 273 (157 males, 59 females, 57 did not indicate their gender; *M*_age_ = 39.57, *SD*_age_ = 12.88; age range = 18–71 years).

Participants originally came from 11 different Syrian towns (the largest group with 27.8% from Ayn El Arab) and most of them found shelter in Urfa (38.8%). The vast majority indicated Islam as their religion (81%; 18.3% did not indicate their religion, less than 1% indicated being Christian or following a different faith) and were either with their partner and their offspring(s) (43.2%) or chose not to provide an answer regarding their family background (31.9%). Nearly half of the participants had been living in a camp for one or two years (45.4%; 38.1% did not answer this question).

#### Procedure

After providing informed consent, participants filled in several questionnaires. With the exception of the Hopkins Symptoms Checklist-25 (HSCL; [39]), for which established Arabic translations exist, all of them were translated and back translated to Arabic from English by two native Arabic speakers and differences in translations were resolved between them.

Participants first competed the 11-items *Regulatory Focus Questionnaire*; (RFQ; [45]), consisting of a 6-item promotion focus subscale (e.g., “How often have you accomplished things that got you psyched to try even harder?”) and a 5-item prevention focus subscale (e.g., “Growing up, did you ever act in ways that your parents thought were objectionable?”–reverse-scored). Items are rated on a 5-point scale (1 = *never or seldom; certainly false* to 5 = *very often; certainly true*). An initial factor analysis indicated that three items loaded on the factor they were not meant to load on. Dropping these items in a further factor analysis rendered a 2-factor solution in line with the original allocation of items to the sub-scales. After Varimax rotation, the promotion factor (4 items) accounted for 25.72% of the total variance, and the prevention factor (4 items) accounted for 15.67% of the total variance. The respective promotion and prevention factor scores were saved as indicators of the respective foci and used in further analyses (rather than computing means). Other research in non-Western cultures has likewise adjusted the RFQ, including dropping of items for reasons of internal consistency [46].

Subsequently, participants’ filled in the 53-item *COPE Scale* [22]. This multidimensional coping inventory assesses the different ways in which people respond to stressful episodes in their lives, with responses given on a 4-point scale (1 = *I usually don’t do this at all* to 4 = *I usually do this a lot*). Five sub-scales (of four items each) measure aspects of problem-focused coping (*M* = 3.02, *SD* = .54; α = .86), namely active coping, planning, suppression of competing activities, restraint coping, seeking of instrumental social support. Four further sub-scales (of four items each) measure aspects of emotion-focused coping (*M* = 3.03, *SD* = .54; α = .82), namely seeking of emotional social support, positive reinterpretation, acceptance, turning to religion. Regarding the last aspect, Syrian refugees have been reported to refer to religious beliefs and practices as a primary source of support [9]. Finally, five further sub-scales (of four items each, except for disengagement by using alcohol or drugs, which is only measured with one item) measure less useful, maladaptive coping (*M* = 2.36, *SD* = .47; α = .72), namely focus on and venting of emotions, denial, behavioral disengagement, mental disengagement, disengagement by using alcohol or drugs. Regarding the last aspect, a recent study among Syrian refugees in Iraq found that approximately half of respondents had more than five alcoholic drinks per week [47]; also, cases of addiction to prescription mediations were reported in several refugee camps [48]. The sub-scale of denial was included in maladaptive (rather than emotion-focused) coping, because in the context of the current study it is not functional to deny one’s situation as a refugee or to act as though the stressor is not real. Indeed, Carver and colleagues ([22], p. 270) point out themselves that the role of denial is “somewhat controversial” as “it only creates additional problems unless the stressor can profitably be ignored. That is, denying the reality of the event allows the event to become more serious, thereby making more difficult the coping that eventually must occur.”

Finally, participants’ psychological symptoms were assessed with the 25-item *Hopkins Symptoms Checklist-25* (HSCL; based on the longer Symptom Checklist, SCL-90; [39]). It comprises two sub-scales (anxiety, 10 items, α = .81; depression, 15 items, α = .87), which are highly correlated (*r* = .73, *p* < .001), and an overall HSCL score (*M* = 2.64, *SD* = .63; α = .91) is usually used. Participants rated how often they experienced particular symptoms in the past week (1 = *not at all* to 4 = *extremely*). Higher scores indicate more severe symptoms. A cut-off point of 1.75 became accepted in refugee settings and in cross-cultural research [49, 50]; a cutoff of 2.0 has been suggested in research on Afghani patients attending primary health care facilities [51]. The Arabic version of the HSCL-25 used here has been found to be reliable and valid among Syrian refugees [17, 52]. The current sample’s mean of 2.64 indicates that on average participants were experiencing clinically significant levels of anxiety and depression.

All data and materials for both samples are available through the Open Science Framework (OSF; http://osf.io/w39hb).

### Sample 2

#### Participants

One hundred eighty-six refugees living in five refugee camps in Germany participated in this research on a voluntary basis. After the administration of the camps provided access, a research assistant approached the refugees in English and with written information in Arabic, informed them about the content of the study, the right to withdraw at any time, and the anonymous treatment of the data. Those who were interested in taking part provided written informed consent before being handed the questionnaires.

Data was collected over a period of two months in the fall of 2015. Ten participants were excluded due to a very high number of missing values and another seven because they were not from Syria. The final sample comprised 169 (152 males, 17 females; *M*_age_ = 27.90, *SD*_age_ = 9.36; age range = 18–68).

Participants originally came from several locations, with the largest groups from Aleppo (17.1%), Damascus (23.2%), and Deir El Zur (9.5%). Data was collected in camps in the Stuttgart region (71%) and in Berlin. The vast majority indicated Islam as their religion (92.3%; 3% indicated to be Christians, others did not indicate their religion or indicated another religion). The majority of the refugees came to Germany alone (67.5%) and 26.1% had at least one family member with them (children, a partner or another close relative). The rest did not respond to this question. Most had arrived in Germany in 2015 (78.1%).

#### Procedure

After providing informed consent, participants completed the same questionnaires as Sample 1. For the *RFQ* [45], factor loadings again did not mirror the original factor structure. After dropping the same three items as in Sample 1 a factor analysis with Varimax rotation using the remaining eight items again led to a 2-factor solution in line with the original allocation of items to the promotion and the prevention sub-scales and with their allocation in Sample 1. The prevention component accounted for 27.2% of the variance and the promotion component accounted for 20.8% of the variance. Factor scores were again saved as indicators of the respective foci and used in further analyses.

The same three sub-scales of the *COPE Scale* [22] as in Sample 1 were formed: problem-focused coping (*M* = 3.33, *SD* = .48; α = .88), emotion-focused coping (*M* = 3.20, *SD* = .44; α = .77), and maladaptive coping (*M* = 2.05, *SD* = .51; α = .80).

Participants’ psychological symptoms were again measured with the *HSCL* ([39]; one item from the depression sub-scale was not assessed). The two sub-scales (anxiety, α = .90; depression, α = .87) were again highly correlated (*r* = .70, *p* < .001) and summarized in one index (*M* = 1.84, *SD* = .55; α = .93).

The data were analyzed using the Statistical Package for Social Sciences (SPSS) v24 (IBM, New York, USA). Direct effects within samples were analyzed using linear regression analyses. Indirect effects within samples (mediation) were analyzed using Hayes’s ([53]; version 2.16) SPSS macro PROCESS for model 4, and indirect effect across samples (moderated mediation) were analyzed using Hayes’s SPSS macro PROCESS for model 8. For further comparisons between samples, t-tests for independent samples were used.

We used ‘mean’ to compute scale means; this process is not sensitive to missing data, but computes means across all non-missing items. In general, data quality was high. On average, there were only 0.13 missing values per person for the items measuring symptoms in the sample from Germany (respectively 0.62 in the sample from Turkey); and there were only 0.29 missing values per person for the items measuring coping in the sample from Germany (respectively 1.68 in the sample from Turkey). Because of this low number of missing values, we did not deem it necessary to apply a substitution procedure for missing values. As would be expected, the data were slightly left skewed (lower scores for the HSCL and maladaptive coping) or right skewed (higher scores for problem- and emotion-focused coping, as well as for promotion and prevention focus). Consequently, the Shapiro-Wilk test was significant for all scales. However, the statistical methods used are robust to violations of the assumption of normal distribution of data and thus appropriate.

## Results

### Sample 1—Syrian refugees in Turkey

To recap, participants completed the RFQ [45], the COPE Scale [22], and HSCL [39]. The current sample’s mean of 2.64 on the HSCL indicates that on average participants were experiencing clinically significant levels of anxiety and depression.

#### Direct effects of regulatory focus on coping and HSCL scores

We regressed participants’ coping strategies and their HSCL scores simultaneously on their promotion and prevention focus factor scores (see Table 1, second column). Both foci were associated with higher levels of problem-focused as well as lower levels of maladaptive coping (in line with predictions 1 and 2). Prevention focus was significantly associated with higher levels of emotion-focused coping, and promotion focus was marginally associated with higher levels of emotion-focused coping. Also, promotion (but not prevention) focus was negatively correlated with anxiety and depression (i.e., higher HSCL scores).

**Table 1:** Direct effects of regulatory focus on coping strategies and HSCL scores in both samples and their moderation by sample origin.

Strategy/Focus	Turkey (*df* = 214)	Germany (*df* = 147)	Moderation by sample (*df* = 359)
Problem-focused coping			
promotion	***B* = .106, *SE* = .033, *t* = 3.21, *p* = .002**	***B* = .196, *SE* = .036, *t* = 5.42, *p* < .001**	*B* = .081, *SE* = .025, *t* = 1.80, *p* = .073
prevention	***B* = .256, *SE* = .033, *t* = 7.76, *p* < .001**	***B* = .096, *SE* = .036, *t* = 2.67, *p* = .008**	***B* = -.080, *SE* = .025, *t* = 3.22, *p* = .001**
Emotion-focused coping			
promotion	*B* = .058, *SE* = .033, *t* = 1.77, *p* = .079	***B* = .107, *SE* = .035, *t* = 3.06, *p* = .003**	*B* = .024, *SE* = .025, *t* = 0.99, *p* = .324
prevention	***B* = .233, *SE* = .033, *t* = 7.04, *p* < .001**	***B* = .096, *SE* = 035, *t* = 2.74, *p* = .007**	***B* = -.068, *SE* = .025, *t* = 2.79, *p* = .006**
Maladaptive coping			
promotion	***B* = -.114, *SE* = .030, *t* = 3.74, *p* < .001**	*B* = -.081, *SE* = .043, *t* = 1.90, *p* = .060	*B* = .016, *SE* = .025, *t* = 0.65, *p* = .519
prevention	***B* = -.075, *SE* = .030, *t* = 2.46, *p* = .015**	*B* = -.055, *SE* = .043, *t* = 1.29, *p* = .198	*B* = .010, *SE* = .025, *t* = 0.39 *p* = .700
HSCL score			
promotion	***B* = -.179, *SE* = .042, *t* = 4.31, *p* < .001**	***B* = -.085, *SE* = .042, *t* = 2.02, *p* = .045**	*B* = .047, *SE* = .030, *t* = 1.55, *p* = .122
prevention	*B* = -.005, *SE* = .042, *t* = 0.13, *p* = .896	*B* = -.055, *SE* = .042, *t* = 1.30, *p* = .194	*B* = -.025, *SE* = .030, *t* = 0.81, *p* = .420

Turkey sample (*N* = 273; coded -1), Germany sample (*N* = 169; coded 1); significant effects are in bold.

#### Indirect effects of regulatory focus via coping on HSCL scores

**Fig 1:**
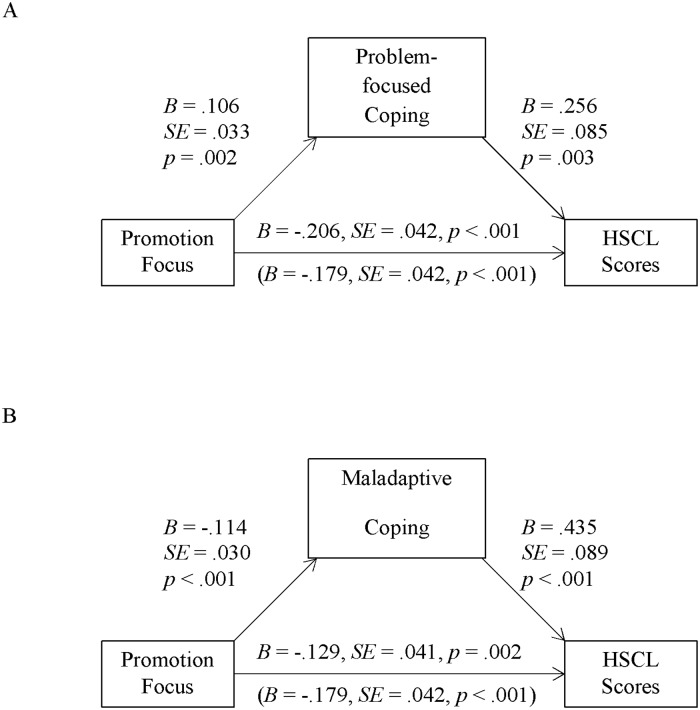
Indirect effects via problem-focused coping (Sample 1, Turkey). Unstandardized regression coefficients for direct and total effects (in parenthesis) of promotion (controlling for prevention) focus factor scores on HSCL scores, as well as the paths via problem-focused coping (Panel A, significant) and maladaptive coping (Panel B, significant) in Sample 1 (*N* = 273).

**Fig 2:**
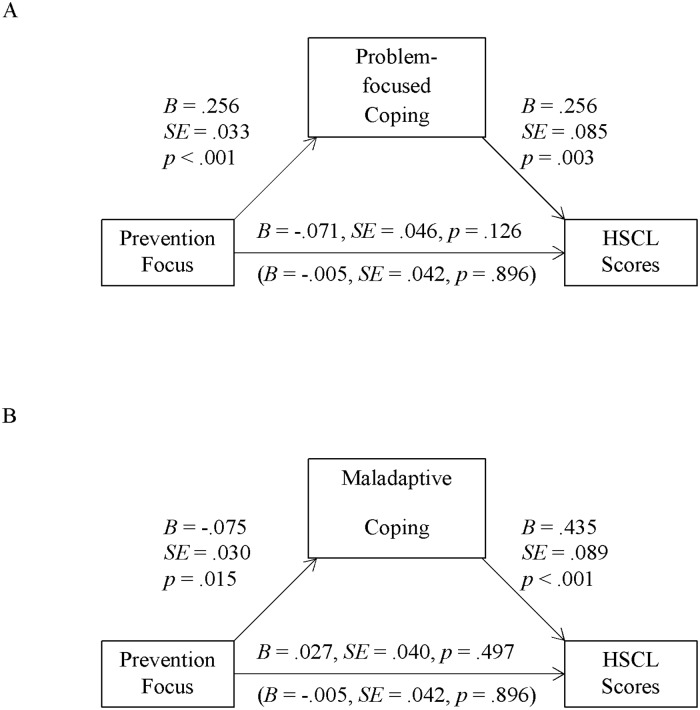
Indirect effects via maladaptive coping (Sample 1, Turkey). Unstandardized regression coefficients for direct and total effects (in parenthesis) of prevention (controlling for promotion) focus factor scores on HSCL scores, as well as the paths via problem-focused coping (Panel A, significant) and maladaptive coping (Panel B, significant) in Sample 1 (*N* = 273).

We tested for indirect effects using the SPSS macro PROCESS by Hayes ([53]; version 2.16, model 4). While testing for the effects of one focus we controlled for the respective other focus. When *problem*-*focused coping* was taken into account as mediator, problem-focused coping correlated positively with promotion focus (as reported above) and with HSCL scores (see Fig 1A). Additionally, there was a positive indirect effect of promotion via problem-focused coping on HSCL scores (see Table 2, second column). According to this indirect effect (and contrary to the total influence) a stronger promotion focus was associated with *higher* HSCL scores via problem-focused coping (see Fig 1A). The parallel analysis for the prevention focus likewise revealed a positive indirect effect (see Table 2, second column): problem-focused coping correlated positively with prevention focus (as reported above) and with HSCL scores (see Fig 2A). Together these paths constitute a significant indirect effect of prevention via problem-focused coping on HSCL scores.

**Table 2:** Indirect effects of regulatory focus via coping strategies on HSCL scores in both samples and their moderation by sample origin.

Strategy/Focus	Turkey	Germany	Index of Moderated Mediation
Problem-focused coping			
promotion	***B* = .027, *SE* = .014, CI [.0073, .0609]**	*B* = .009, *SE* = .022; CI [-.0351, .0525]	***B* = .015, *SE* = .010, CI [.0010;.0443]**^a^
prevention	***B* = .066, *SE* = .024, CI [.0211, .1190]**	*B* = .004, *SE* = .011; CI [-.0159, .0309]	***B* = -.028, *SE* = .015, CI [-.0653;-.0058]**
Emotion-focused coping			
promotion	*B* = .001, *SE* = .008, CI [-.0005, .0311]	*B* = .016, *SE* = .012, [-.0033; .0481]	*B* = .007, *SE* = .008, CI [-.0040;.0298]
prevention	*B* = .039, *SE* = .022, CI [-.0083, .0834]	*B* = .010, *SE* = .011; CI [-.0066, .0398]	***B* = -.021, *SE* = .012, CI [-.0524;-.0024]**
Maladaptive coping			
promotion	***B* = -.050, *SE* = .016, CI [-.0903, -.0250]**	***B* = -.041, *SE* = .019, CI [-.0820, -.0068]**	*B* = .015, *SE* = .022, CI [-.0271;.0693]
prevention	***B* = -.033, *SE* = .014, CI [-.0623, -.0074]**	*B* = .014, *SE* = .012; CI [-.0032, .0450]	*B* = .009, *SE* = .022, CI [-.0344;.0564]

Turkey sample (*N* = 273; coded -1), Germany sample (*N* = 169; coded 1); significant effects are in bold; confidence intervals at 95%.

^a^ The sign of this moderated mediation index should be negative given the indirect effects in the analysis for the separate samples. However, due to the substantive mean differences between both studies (especially regarding promotion focus) the conditional indirect effects are reversed in the overall analysis. Nonetheless, this analysis provides evidence for a difference regarding the indirect effect between both samples.

In sum, both foci were associated with higher HSCL scores via problem-focused coping: Both were positively associated with problem-focused coping (in line with prediction 1), which in turn was positively associated with HSCL scores (contrary to prediction 1). This might seem to contradict the idea that promotion focus constitutes a resilience factor. However, its strong direct effect on *reduced* HSCL scores reported above clearly shows that it is. At the same time, this effect is to some extent counteracted by the fact that promotion is also associated with increased problem-focused coping, which–in the specific current context and contrary to its general consideration in the coping literature–is detrimental to wellbeing. In other words, whilst the direct effect of promotion was adaptive for mental health (i.e., lower HSCL scores), its indirect effect via increased problem-focused coping was detrimental–not because of promotion per se, but because of its association with increased problem-focused coping. We return to this point in the discussion.

Regarding *emotion-focused coping* as a mediator, no indirect effect of either focus on HSCL scores emerged (see Table 2, second column).

Finally, for *maladaptive coping*, the direct negative effect of promotion on HSCL scores remained significant when including this mediator in the analysis (see Fig 1B). Promotion focus correlated negatively with maladaptive coping, and this coping style positively predicted higher HSCL scores. As result, promotion focus asserted an indirect negative effect on HSCL scores via maladaptive coping (in line with prediction 2; see Table 2, second column): the stronger their promotion focus was, the less symptoms of anxiety and depression refugees reported because of less maladaptive coping. Turning to prevention focus, after taking maladaptive coping into account its direct effect on HSCL scores remained non-significant (Fig 2B). Similar to promotion focus, prevention focus correlated negatively with maladaptive coping, which–as indicated above–correlated positively with HSCL scores. Consequently, prevention focus also asserted a negative indirect effect on HSCL scores via maladaptive coping (in line with prediction 2; see Table 2, second column). Thus, both foci predicted a reduction in HSCL scores via reduced maladaptive coping.

Overall, among Syrian refugees in Turkey both foci were associated with increased symptoms of anxiety and depression via augmented problem-focused coping, but also served as a buffer for these symptoms via reduced maladaptive coping. These effects cancelled each other out for prevention focus (which thus did not assert a total effect on HSCL scores). Importantly, promotion focus was directly and positively related with refugees’ wellbeing (i.e., less symptoms), partly based on its indirect effect via maladaptive coping.

### Sample 2—Syrian refugees in Germany

To recap, participants completed the same scales as Sample 1. The analyses below followed the same strategy as in Sample 1.

#### Direct effects of regulatory focus on coping and HSCL scores

We again regressed participants’ coping strategies and HSCL scores simultaneously on their promotion and prevention factor scores (see Table 1, column 3). Both foci positively predicted problem-focused (in line with prediction 1) and emotion-focused coping. Maladaptive coping was marginally and negatively correlated with promotion focus, but not with prevention focus (partially in line with prediction 2). Finally, participants’ HSCL scores were negatively associated with participants’ promotion but not prevention focus. In sum, both foci were again associated with higher levels of problem- and emotion-focused coping. However, only promotion focus was associated with lower levels of maladaptive coping and lower HSCL scores.

#### Indirect effects of regulatory focus via coping on HSCL scores

**Fig 3:**
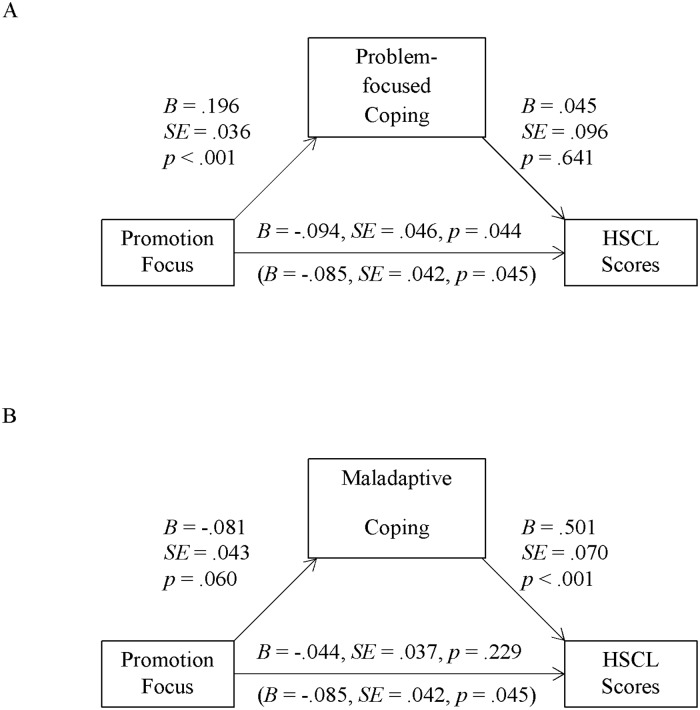
Indirect effects via problem-focused coping (Sample 2, Germany). Unstandardized regression coefficients for direct and total effects (in parenthesis) of promotion (controlling for prevention) focus factor scores on HSCL scores, as well as the paths via problem-focused coping (Panel A, not significant) and maladaptive coping (Panel B, significant) in Sample 2 (*N* = 169).

**Fig 4:**
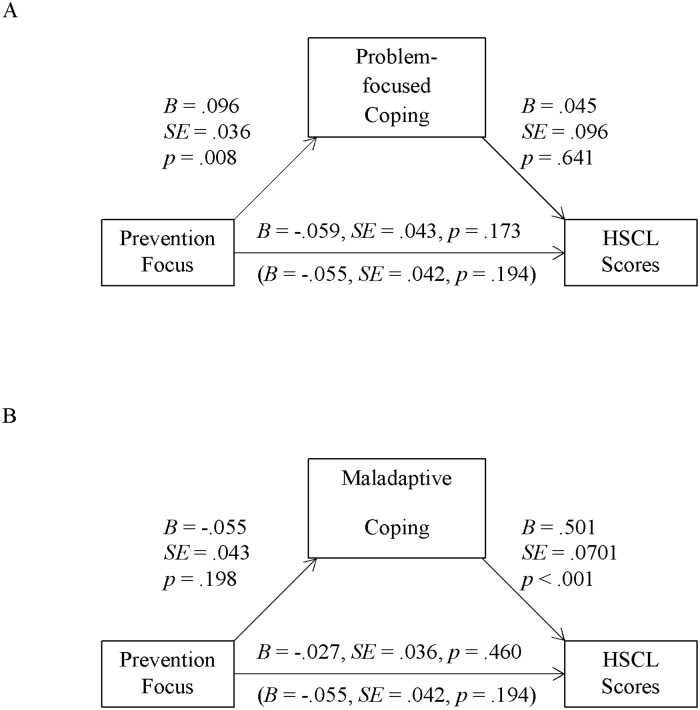
Indirect effects via maladaptive coping (Sample 2, Germany). Unstandardized regression coefficients for direct and total effects (in parenthesis) of prevention (controlling for promotion) focus factor scores on HSCL scores, as well as the paths via problem-focused coping (Panel A, not significant) and maladaptive coping (Panel B, not significant) in Sample 2 (*N* = 169).

We again tested for indirect effects of both foci (controlling for the respective other focus) using the SPSS macro PROCESS by Hayes ([53] model 4). Neither problem-focused nor emotion-focused coping carried an indirect effect of promotion or prevention focus on HSCL scores (contrary to prediction 1; see Table 2, third column; for problem-focused coping and comparison to Sample 1, see also Figs 3A and 4A). Furthermore, there was no evidence for an indirect effect of prevention focus via maladaptive coping on HSCL scores (contrary to prediction 2; see Table 2, third column, and Fig 4B). However, promotion focus predicted HSCL scores via maladaptive coping (see Table 2, third column): promotion focus correlated with less maladaptive coping and this coping strategy positively correlated with HSCL scores (in line with prediction 2; see Fig 3B).

Overall, and as in Sample 1, promotion focus was directly associated with reduced symptoms of anxiety and depression, and also indirectly via a reduction in maladaptive coping. Unlike Sample 1, promotion focus had no indirect effect via problem-focused coping. Also differing from the sample in Turkey, prevention focus seemed to be of less importance in the context of coping for the sample in Germany. To flesh out these and further comparisons, we now turn to a set of analyses across both samples.

### Analyses comparing both samples

To draw comparative conclusions across samples and thus locations, we employed three different analysis strategies. First, we tested for sample differences in scale means using t-tests for independent samples. Second, we tested whether the direct effects of regulatory focus on coping and symptoms of anxiety and depression differed between samples using multiple regression analyses with standardized promotion focus, prevention focus, sample origin (coding: Turkey -1, Germany 1) and the foci by sample interactions as predictors. Finally, we tested whether sample origin moderates the effect of the respective regulatory foci via coping styles on symptoms of anxiety and depression with moderated mediation analysis, using Model 8 of the PROCESS macro by Hayes [53]. The second and third sets of comparative analyses are highly redundant with the analyses reported separately for the studies above. We therefore only report the test for the moderation of the respective effects by sample (see Table 2, fourth column, and Table 3, fourth column), crucial for the comparison between countries.

**Table 3:** Comparisons of regulatory focus, coping strategies and HSCL scores between samples.

	Turkey	Germany	*t* (*p*)	*d*
Prevention Focus	3.89 (0.94)	3.84 (1.00)	0.53 (.596)	0.05
Promotion Focus	3.10 (0.81)	3.81 (0.86)	8.72 (< .001)	0.85
Problem-focused coping	3.02 (0.54)	3.33 (0.48)	6.15 (< .001)	0.60
Emotion-focused coping	3.03 (0.54)	3.20 (0.44)	3.55 (< .001)	0.34
Maladaptive coping	2.36 (0.47)	2.05 (0.51)	6.54 (< .001)	0.63
HSCL scores	2.64 (0.63)	1.84 (0.55)	13.67 (< .001)	1.36

Turkey sample (*N* = 273), Germany sample (*N* = 169).

#### Differences between groups

Whilst the samples did not differ in their prevention focus, the sample in Germany had a stronger promotion focus (for all results, see Table 3). However, this difference should be interpreted with caution, because it relies on means of the items loading on the promotion factor rather than factor scores (as factor scores are not suitable for t-tests, given that they have a mean of zero in each sample). All following reported analyses are based on factor scores. The sample in Germany also reported more problem-focused and emotion-focused coping, as well as less maladaptive coping. Finally, HSCL scores were substantially higher in the Turkey sample. This difference is dramatic according both to the large effect size and the mean of the Turkey sample being clearly above the clinical cut-off value.

#### Moderation of direct effects of regulatory focus on coping and HSCL scores

In both samples promotion focus was associated with more problem-focused coping. This relation was marginally stronger among refugees in Germany than in Turkey (for all results, see Table 1, column 4). There were no other differences between samples regarding direct relations between promotion focus and other considered variables. A stronger prevention focus also predicted more problem-focus coping in both samples. This effect was, however, stronger in the Turkey than in the Germany sample. The same pattern occurred for the relation between prevention focus and emotion-focused coping, namely a stronger positive correlation among refugees in Turkey (vs. Germany). For maladaptive coping and HSCL scores, sample origin did not moderate their relation with prevention focus.

#### Moderation of indirect effects of regulatory focus via coping on HSCL scores

The findings mirror those for the direct effects reported above. For problem-focused coping, a positive indirect effect of both foci on HSCL scores occurred only in the Turkey sample. These differences were statistically significant (in line with prediction 3; for all results, see Table 2, column 4). There was also a difference regarding the indirect effect of prevention focus via emotion-focused coping on HSCL scores that was somewhat surprising, given that this indirect effect occurred in neither of the samples when considered individually. However, a stronger prevention focus predicted higher HSCL scores (via emotion-focused coping) in the Turkey sample to a stronger extend than in the Germany sample. This difference is an outcome of a) the stronger statistical power in the analysis across *both* samples and b) the large standard error of the effect in the Turkey (*SE* = .022) compared to the Germany sample (*SE* = .011), which most likely prevented the detection of an indirect effect in this sample when considered individually.

## Discussion

The present work responds to recent calls regarding the need to investigate resilience and coping strategies amongst refugees [8, 9, 12–15]. In doing so, it drew on the notion that personality, and in particular individual’s regulatory focus influences coping and in turn adjustment [21, 25, 28]. Consequently, it explored direct and indirect relations between regulatory focus, coping strategies, and mental wellbeing in displaced Syrian refugees living in Turkey and Germany. Several findings emerged from this endeavor.

First, compared to the sample of Syrian refugees in Germany, among Syrian refugees in Turkey symptoms of anxiety and depression (measured with the HSCL) as well as levels of maladaptive coping were higher, whilst levels of problem- and emotion-focused coping were lower. Moreover, the HSCL score in the Turkey sample was well above the clinical cut-off point, stressing the urgent need for interventions among these refugees. It also suggests that Syrian refugees in Turkey experience the situation as more severe, which should impact coping responses and outcomes [24, 42]. Indeed, problem-focused coping, generally understood to be adaptive [21, 22], was detrimental to wellbeing in the Turkey but not the Germany sample. Adding to this, in the Turkey sample not only promotion focus, but also prevention focus increased symptoms of anxiety and depression indirectly via more problem-focused coping. This suggests the stressor being perceived as uncontrollable, a situation in which disengagement or emotion-focused coping are more adaptive than problem-focused coping [18, 24, 41, 54]. The fact that also prevention focus was associated with the, in this particular context, disadvantageous strategy of problem-focused coping dovetails with work showing that prevention focus can be associated with risky choices under situations of loss [55]. Taken together, the current findings indicate a much higher need for interventions targeting mental wellbeing in Turkey (compared to Germany) and suggest that reducing maladaptive may be a promising strategy. Furthermore, future research could explore if interventions to direct problem-focused coping to *specific* and *attainable* outcome (within the given the context) reverse its detrimental effect found here. Finally, enhancing emotion-focused coping beyond a critical threshold might likewise result in positive effects for mental wellbeing.

Second, in both samples maladaptive coping increased symptoms of anxiety and depression, in line with the notion that it is dysfunctional [21, 22]. Interventions should thus also target maladaptive coping and draw attention to its negative consequences: Whilst it might provide short-term alleviation, it undermines long-term mental wellbeing. Targeting first and foremost maladaptive (rather than problem-focused) coping seems warranted, as the latter was negatively associated with wellbeing in both samples.

Finally, and most importantly, in both samples promotion focus emerged as a resilience factor: It was directly associated with reduced symptoms of anxiety and depression in Syrian refugees. Also in both samples, promotion was more strongly and consistently associated with a reduction in maladaptive coping than prevention focus. This finding dovetails with research indicating that promotion entails a focus on gains [36], a preference for upward counterfactuals [37], and is associated with optimism (and, in turn, better mental health; [25, 32, 43]). In both samples a promotion focus was also associated with lower symptoms of anxiety and depression, in line with recent research showing that across different layers of personality (i.e., traits, life goals, and life stories) promotion (compared to prevention) focus is related to better psychological and physical health [56]. In the current context, further reasons for this positive influence of promotion focus might lie in its association with openness to change (vs. an association of prevention focus with a preference for stability; [57]) and that loss is experienced more severely in a prevention than in a promotion focus [58]. Future research might explore how regulatory focus shapes dimensions of the cognitive representation of the stressor as proposed by the common-sense model of self-regulation in coping with illness [23, 44]. Alternatively, regulatory focus may be a further dispositional moderator (alongside optimism and perfectionism) of the relation between stressor representations and coping strategies [24]. Regulatory focus can be found at the individual and the group level and varies chronically and situationally [45, 59]. A final intervention recommendation from the above findings is that instilling a promotion focus in refugees can be expected to have beneficial consequences.

### Limitations

There are several caveats that come with our findings. First and foremost, our samples are not representative of the groups of Syrian refugees in Turkey or Germany, which limits the generalizability of the current findings. Having representative samples would obviously be desirable, but at the time of data collection, not even the exact number of refugees in both countries was known to government officials and detailed statistics were non-existent. We aimed at collecting data from heterogeneous samples by approaching refugees in various sites both in Turkey and in Germany, and within Germany in states with different policies. Nonetheless, the current data can make no claims regarding representativeness.

Additionally, and in relation to the above point, all differences between samples could either stem from differences between the refugees or the different conditions they are faced with. The demographics of our samples and media reports suggest that those who travel on from Turkey to Europe are on average younger, have a higher SES, and are male rather than female. At the same time, refugees in Turkey continue to witness the direct or indirect consequences of the conflict due to their proximity to Syria. Also, unlike Germany, Turkey does not grant Syrians refugee status along with legal rights, but only temporary asylum seeker status. To this point, a recent study found higher HSCL scores and increased worry as well as social withdrawal among refugees under restrictive (vs. supportive) immigration policies [60]. There are a host of further factors that could be driving effects related to sample differences, stemming from differences between the host countries (e.g., individualism/collectivism or income per capita) as well as different experiences of the refugees in these countries (e.g., perceived stress or experience of traumatic events). Future research is clearly needed to also explore their role in impacting coping and wellbeing. The current data does not allow identifying the causes of sample differences. They do, however, allow comparing whether effects of self-regulation via coping styles on wellbeing hold across both samples and identifying sample differences in these relations.

Further limitations are shared by other research on mental health and wellbeing in Syrian refugees [8, 9, 12, 61]. For example, cross-sectional designs do not allow for claims regarding causality and rather small, non-random convenience samples limit generalizability. Furthermore, additional factors known to impact both coping and wellbeing were not taken into account (e.g., length of displacement, previous psychological conditions). Also, the assessment of clinical symptoms is more accurate and appropriate when integrating local modes of expressing distress and understanding symptoms [62–64]. Finally, the RFQ has not been validated in refugee populations and influences of language and cultural differences cannot be ruled out [65].

### Contributions

Nevertheless, the present work makes several contributions to research on mental health and wellbeing of Syrian refugees, the coping literature, and the literature on regulatory focus. It is among the first research investigating resilience factors and coping among Syrian refugees. Recent work found that social identification is one such resilience factor. Specifically, discrimination was not associated with poorer mental and physical health for Syrian refugees in Turkey who derived a sense of efficacy from their Syrian identity; and it was especially associated with lower depression and anxiety for those who derived a sense of belonging from their identity [17]. The current work suggests that differences in self-regulation constitute a further source of resilience, with a promotion focus serving a buffering function and maladaptive coping exacerbating negative effects on mental wellbeing.

The current findings also contribute to and extend the coping literature. First, research has found problem-focused coping with trauma to be associated with better [66] and worse [67] psychological outcomes. It has been suggested that the uncontrollability of a stressor might render problem-focused coping ineffective [24, 68] and that when there is no alternative to take up, continuous commitment to an unattainable goal constitutes a severe form of distress [41, 43]. In the current work problem-focused coping was either not (Sample 2) or negatively (Sample 1) associated with psychological wellbeing, highlighting the fact that indeed it is impossible to determine the general (mal)adaptiveness of any way of coping [20]. Second, research on how individual differences and personality affect coping [21] has mainly focused on the Big Five personality traits [69] and optimism [30]. The current work extends this perspective to self-regulation and the notion that how people appraise the world (“ways of seeing”) and their behavioral strategies in it (“ways of coping”) are both impacted by regulatory focus [28]. As such, it replicates and expands the scarce literature on regulatory focus and coping in undergraduates [25] to severely stressful situations.

### Conclusion

Overall, the current work provides one of the very few psychological understandings of Syrian refugees’ coping and mental health. The findings suggest that effective interventions for Syrian refugees that aim to target resilience and coping would be advised to aim at reducing maladaptive coping (i.e., not dealing with the stressor or its associated distress) and to instill an orientation on nurturance, growth and gains (i.e., a promotion focus). Though generally considered effective, problem-focused coping (dealing with the stressor to remove it or to diminish its impact) had a negative (Turkey) or no impact (Germany) on mental wellbeing.
